# Exploring the physiological plasticity of giant grouper (*Epinephelus lanceolatus*) to dietary sulfur amino acids and taurine to measure dietary requirements and essentiality

**DOI:** 10.1007/s10695-023-01222-4

**Published:** 2023-07-28

**Authors:** Caroline Lourdes Candebat, Thibault Eddie, Adrien Francois Marc, Fernando Fernando, Leo Nankervis

**Affiliations:** 1 Centre for Sustainable Tropical Fisheries and Aquaculture & College of Science and Engineering, James Cook University, QLD, Townsville, 4811 Australia; 2 Department of Biotechnology and Food Science, Norwegian University of Science and Technology (NTNU), 7491 Trondheim, Norway

**Keywords:** Aquaculture, Fish nutrition, Histopathology, Intestinal health, Amino acid interaction

## Abstract

Giant grouper (*Epinephelus lanceolatus*) is an economically important yet under-researched species, still reliant on ‘trash fish’ or generic aquafeeds. The transition toward sustainable formulations is contingent on establishing requirements of target species for limiting nutrients, among which the sulfur amino acids (methionine and cysteine) commonly limit fish growth. Further, there remains significant conjecture around the role of the sulfonic acid taurine in marine aquafeed formulation and its relationship to sulfur amino acids. To develop a species-specific feed formulation for giant grouper, dietary methionine was modulated in a dose-response experiment to achieve five graded levels from 9.5 to 21.5 g/kg, including an additional diet with methionine at 18.6 g/kg supplemented with 8 g/kg taurine. The mean (±SD) cysteine level of the diets was 4.5 ± 0.3 g/kg. Each diet was randomly allocated to triplicate tanks of 14 fish (83.9 ± 8.4 g). The best-fit regression for growth showed that the optimal dietary methionine content was 15.8 g/kg and the total sulfur amino acid content was 20.3 g/kg. Inadequate dietary methionine content triggered physiological responses, including hepatic hyperplasia and hypoplasia at 9.5 and 21.5 g/kg, respectively, and high aspartate transaminase levels at 18.9 g/kg. Moreover, inadequate dietary methionine contents resulted in higher densities of mixed goblet cell mucin and reduced absorptive surface area of posterior intestinal villi. Our results suggest that adequate levels of methionine, but not taurine, improved posterior intestinal conditions and liver homeostasis. These findings may aid in formulating aquafeeds to optimize gastrointestinal and liver functions in juvenile giant grouper.

## Introduction

The optimal supply of essential nutrients is critical to maintain and improve the health, growth, stress tolerance, and physiological homeostasis of farmed, carnivorous fish (Trichet 2010). Concomitant with the global expansion and intensification of aquaculture, fish nutrition is transitioning from ‘trash’ fish and fish meal and toward pelleted aquafeeds that, to date, include a multitude of proteins of terrestrial animal and plant origin (Béné et al. 2016). Utilizing diverse protein sources is critical to aquafeeds’ economic and sustainable development. However, detailed knowledge of optimal nutrient requirements for growth and normal physiological function is required as essential amino acid composition and digestibility of protein source vary (Jia et al. 2013). For instance, plant proteins are high in fiber, carbohydrates, and antinutrients which are often attributed to alterations in gut and liver functions (Jia et al. 2013) and, consequently, plant proteins often place pressure on digestive and absorptive capacities, which are dictated by anatomical and physiological traits of the tissue. Receiving less research attention, nutrient imbalances caused by varying raw material composition and bioavailability may also be detrimental to organ function (Li et al. 2021). However, the increasingly cost-effective manufacturing of synthetic amino acids, such as methionine and lysine, promotes the formulation of aquafeed tailored to species-specific requirements for optimized physiology (Leuchtenberger et al. 2005; Nunes et al. 2014; Selle et al. 2020). These tailored and nutrient-based formulations of aquafeed require estimations of nutrient requirements and raw material digestibility to promote growth while maintaining fish health and well-being (Jobling 2016).

Methionine (Met) is one of the key limiting amino acids in protein sources. Fish fed Met at sub- and supra-optimal levels undergo significant metabolic shifts toward protein catabolism and reduced protein turnover, impairing organ function and growth (Candebat et al. 2021; Li et al. 2021; Rolland et al. 2015). Assimilated Met is trans-sulfurated to endogenous cysteine (Cys) (Candebat et al. 2021; Griffin et al. 1994; Harding et al. 1977; Zehra and Khan 2016), which intracellular availability is also imperative for fish welfare. Cys forms substrate for the synthesis of various functional metabolites in addition to its role as building block of peptides and proteins (Ball et al. 2006; Coloso et al. 2006; Serpa 2020). The metabolically interactive sulfur amino acids (SAAs), Met, and Cys, as well as the amino-sulfonic acid taurine (Tau), form a comprehensive nutritional requirement in animals with shared and individual functions (Andersen et al. 2016; Brosnan and Brosnan 2006; Candebat et al. 2020). Tau is a critical nutrient for several marine carnivorous species and is often lacking in proteins from non-animal origin. Dietary Tau has multiple functions, including maintaining hepatic homeostasis by (1) conjugating with hepatic cholesterol derivatives to form bile salts, emulsifying ingested lipids (Kim et al. 2008); (2) conjugating with hepatic bilirubin, removing toxic by-products of heme breakdown (Goto et al. 2001; Sakai et al. 1987); (3) regulating glucose metabolism (Zhang et al. 2019); and (4) contributing to cell osmolality (Takagi et al. 2006). Information on the interactive effects of dietary Met, Cys, and Tau on fish physiology is still limited (Krogdahl et al. 2020; Li et al. 2021; Nordrum et al. 2000). Dietary Met and Cys can meet the requirement for SAAs, commonly referred to as the total sulfur amino acid (*TSAA*) requirement. While dietary Met can meet the entire requirement for sulfur amino acids, its bioconversion to endogenous cysteine is unidirectional, meaning that dietary cysteine can only meet the component of the *TSAA* requirement specific to Cys and its downstream metabolites. In fish, Cys can contribute up to 60% of the *TSAA* requirement (Ball et al. 2006; Candebat et al. 2021), contributing to the incorporation of cysteine into proteins or peptide, or downstream metabolic processes such as the formation of taurine or glutathione (Yin et al. 2016). In contrast to the limited utility of Cys, Met can be bioconverted to all metabolites of the *TSAA* group. Therefore, the *TSAA* requirement may be described as the amount of dietary Met needed to meet the requirement at low and consistent dietary cysteine inclusion.

Giant grouper (*Epinephelus lanceolatus*), hereafter referred to as GG, is an economically important aquaculture species in Asia and increasingly popular in Australia due to its rapid growth, high value, and meat quality (Dennis 2020; Dennis et al. 2020). Yet, the culture of this carnivorous and high-value species relies on trash fish and generic aquafeeds (Nankervis et al. 2022; Rimmer and Glamuzina 2017). Aquafeeds, currently used for *Epinephelus* spp., range in protein content from 44 to 62% (Nankervis et al. 2022) and often contain proteins of animal and plant origin that lack essential amino acids or are not readily accessible due to their low digestibility (Halver 2002; Kaushik and Seiliez 2010). Anecdotal reports have recorded enlarged livers in GG fed generic marine aquafeed (Nankervis et al. 2022), indicating nutritional imbalances. The histological implications of these occurrences are under-studied. In several *Epinephelus* spp., dietary Tau supplementation benefited production, health, and welfare by improving energy utilization and amino acid uptake; promoting the synthesis of protein, lipid, and purine; and accelerating growth (Shen et al. 2019). Further, Tau supplementation increased the lipid digestibility and growth of GG fed diets containing high levels of soya bean meal (Lin and Lu 2020). However, Tau enhanced intestinal digestive functions and regulated the glycolipid metabolism in hybrid grouper (*E. fuscoguttatus* × *E. lanceolatus*) (Qian et al. 2021). While taurine has important physiological functions, it is not clear whether grouper have a dietary taurine requirement or if its role can be met by the endogenous bioconversion of sulfur amino acids. In juvenile hybrid grouper, Li et al. (2020) quantified the requirement for Met at 14.5-g/kg diet at a constant Cys level of 6.9 g/kg. While this provides a starting point for quantifying requirements for GG, initial estimates indicate that amino acid requirements for GG vary from hybrid grouper (Nankervis et al. 2022) and that effective formulation of feeds for the industry requires species-specific requirement values, expressed on a digestible nutrient basis. Further, a thorough evaluation of histological structures and biochemistry of Met-, Cys-, and Tau-active tissues may provide insight into the interactions between fish nutrition and tissue physiology, the ramifications of which control tissue function, nutrient requirements, and, ultimately, fish growth responses.

Thus, this study had the following goals: (1) quantify the *TSAA* requirement on a dietary and digestible basis, (2) assess Tau’s effectiveness in promoting the growth and well-being of GG at dietary levels that meet the *TSAA* requirement, and (3) use quantitative histomorphological and chemical assessments of hepatic and intestinal tissues to elucidate the effects of adequate and inadequate dietary *TSAA* and taurine.

## Materials and methods

### Ethics statement

The experiment of this study was approved by the James Cook University’s Animal Ethics Committee, Townsville, Australia (Approval number: A2713).

### Experimental design and diets

The *TSAA* requirements and effect of dietary Tau in a SAA replete diet on the performance of GG were quantified by conducting a feeding trial that applied a factorial dose-response design. Six isoproteic (579.7 ± 0.34 g/kg) and isoenergetic (19.08 ± 0.45 MJ/kg) diets were formulated to contain either one of five incrementally increasing levels of Met (9.5, 11.6, 14.0, 17.9, and 21.5 g/kg) at a Tau content of 1.8 ± 0.05 g/kg. These supplemental amino acids replaced glycine on an isonitrogenous basis. An additional diet was formulated to contain Met at 18.6 g/kg with a dietary Tau level of 8.0 g/kg (Tables 1 and 2). All seven diets had a constant level of Cys at 4.5 ± 0.12 g/kg.

**Table 1:** Raw material composition and digestibility of experimental diets formulated to contain six increasing levels of dietary methionine and two levels of dietary taurine at a constant level of dietary cysteine

	Diet					
	D1	D2	D3	D4	D5	D6 + T
Ingredients (g/kg dry matter)						
Fish meal, 65% CP^a^	250	250	250	250	250	250
Soy protein concentrate, Selecta60^a^	210	210	210	210	210	210
Wheat flour^d^	200	200	200	200	200	200
Gelatin^b^	100	100	100	100	100	100
Faba beans^a^	55	55	55	55	55	55
Lupin seed meal, 49% CP^a^	50	50	50	50	50	50
Fish oil, anchovy^a^	50	50	50	50	50	50
Blood cell meal^a^	47	47	47	47	47	47
Canola oil	14.5	14.5	14.5	14.5	14.5	14.5
L-lysine^a^	3.5	3.5	3.5	3.5	3.5	3.5
Vitamin mix^a,e^	1.0	1.0	1.0	1.0	1.0	1.0
Mineral mix^a,f^	1.0	1.0	1.0	1.0	1.0	1.0
Vitamin E-50^a^	0.2	0.2	0.2	0.2	0.2	0.2
Glycine^a^	13.3	10.9	9.1	6.3	1.7	2.2
Diatomaceous earth	4.5	4.3	4.1	3.9	3.4	3.0
DL-methionine^a^	-	2.6	4.6	7.6	12.7	7.6
Taurine^c^	-	-	-	-	-	5.0
Yttrium	1.0	1.0	1.0	1.0	1.0	1.0
Proximate analysis (g/kg DM, unless stated otherwise)						
Dry matter (%)	90.6	88.4	86.4	91.3	87.3	91.7
Ash	72.6	74.3	75.1	74.3	72.7	72.5
Total lipid	99.3	99.6	99.8	96.0	98.2	91.5
Total protein	572.0	569.9	583.2	578.0	583.0	592.4
Carbohydrate	256.1	256.2	242.0	251.7	246.2	243.7
Gross energy (MJ/kg)	19.5	19.1	18.8	19.7	19.1	18.3
Apparent digestibility coefficient (%)						
Total lipid	92.0	93.2	93.0	92.5	92.9	92.4
Total protein	83.5	91.4	89.5	87.0	87.8	89.5
Carbohydrate	23.7	37.2	19.4	12.1	14.7	21.9
Methionine	88.5	95.9	93.9	93.1	94.2	94.7
Cysteine	60.9	77.3	56.5	54.2	53.9	65.3

^a^Skretting Australia, Cambride, Tasmania

^b^Collagenx, Australia

^c^Bulk Nutrients, Grove, Tasmania, Australia

^d^Woolworth essentials, Australia

^e^Composition (g/kg unless otherwise stated): biotin, 1; folic acid, 5; niacin, 45; pantothenic acid, 10; pyridoxine, 10; riboflavin, 20; thiamin, 10; vitamin B12, 0.05; vitamin C, 150; vitamin A, 3000 IU/g; vitamin D, 2400 IU/kg; vitamin K (menadione), 10; inositol, 250; antioxidant, 15

^f^Composition (g/kg): magnesium, 59.4; copper, 1; iron, 8; manganese, 5; selenium, 0.02; zinc, 20; iodine, 0.8; cobalt, 0.1; ash, 700; moisture, 20

**Table 2:** Amino acid composition (g/kg DM) and total sulfur amino acid contents (g/kg DM) of experimental diets

	Diet					
	D1	D2	D3	D4	D5	D6 + T
*Essential amino acid contents*						
Arginine	31.2	35.3	33.1	33.9	30.7	34.3
Histidine	12.0	13.1	13.1	12.5	11.8	14.0
Isoleucine	15.8	16.2	17.1	17.3	15.9	17.8
Leucine	34.2	34.0	34.6	36.0	33.0	36.8
Lysine	29.1	33.2	32.8	33.0	29.2	31.6
Methionine	9.5	11.6	14.0	17.9	21.5	18.6
Phenylalanine	23.9	22.3	23.2	24.3	22.3	25.6
Threonine	20.0	19.7	20.2	21.5	19.2	21.2
Valine	21.4	20.7	22.2	22.8	20.7	22.7
*Non- and semi-essential amino acids and derivative contents*						
Alanine	32.4	31.4	32.8	33.9	31.9	34.4
Aspartic acid	48.0	47.6	47.8	49.7	46.8	51.2
Cysteine	4.4	4.3	4.3	4.7	4.2	4.9
Glutamic acid	78.3	78.5	80.4	83.5	78.0	87.2
Glycine	62.7	55.4	50.5	55.6	49.2	54.1
Proline	34.5	34.0	34.8	35.7	33.0	37.7
Serine	24.2	24.5	24.8	25.8	23.8	27.0
Tyrosine	14.9	14.2	14.7	15.5	14.5	15.7
Taurine	1.8	1.7	1.7	1.9	1.7	8.0
*TSAA contents*						
∑ *TSAA* [Met + Cys]	13.9	15.9	18.3	22.6	25.7	23.5
∑ *TSAA* [Met]	14.9	16.9	19.3	23.6	26.6	24.7

For the manufactured diet, dry ingredients larger than 1 mm in particle sizes, such as lupin meal, soybean meal, and faba beans, were finely milled through a hammer mill (Thomas-Wiley, PA, USA), fitted with a 1-mm screen. All dry ingredients were mixed in a cement mixer for 1.5 h, forming the basal diet, and split into six equal parts. Each part was supplemented with crystalline DL-Met, Tau, L-glycine, and diatomaceous earth according to Table 1 and separately mixed in a Hobart 120A Planetary Mixer (Troy Pty Ltd., OH, USA) for 15 min. During separate mixing, yttrium was added accordingly to the dry mesh to allow for digestibility measurements (Tables 3 and 4). Following mixing, oil and water were slowly added to the dry mesh while mixing continued, forming a moist dough. The dough of even consistency was then further processed through a Hobart A120 with a mincing attachment fitted with a 5-mm die. Pellets were then steamed at 100 °C for 10 min and oven-dried at 60 °C to a moisture content between 8.3 and 13.6% before being stored at −18 °C until further use.

**Table 3:** Apparent digestibility coefficients (%) for amino acids of each experimental diet

	Diet					
	D1	D2	D3	D4	D5	D6 + T
*ADC of essential amino acid*						
Arginine	93.5	97.9	95.1	92.9	93.4	94.3
Histidine	87.8	95.7	90.3	87.3	87.5	90.6
Isoleucine	87.0	94.9	89.9	88.6	87.9	90.6
Leucine	88.4	95.2	90.6	88.9	88.7	91.4
Lysine	89.4	96.3	92.7	90.8	91.0	92.2
Methionine	88.5	95.9	93.9	93.1	94.2	94.7
Phenylalanine	88.8	95.2	90.8	89.3	89.4	91.9
Threonine	85.6	93.5	86.1	85.7	84.7	88.4
Valine	87.3	94.5	89.4	87.4	86.9	90.2
*ADC of non- and semi-essential amino acids and derivative*						
Alanine	89.4	95.3	90.3	89.2	89.6	91.6
Aspartic acid	84.3	92.3	84.8	82.7	84.0	86.4
Cysteine	60.9	77.3	56.5	54.2	53.9	65.3
Glutamic acid	88.7	95.5	91.1	89.6	90.0	92.0
Glycine	91.5	95.3	89.4	88.8	88.2	91.0
Proline	90.6	96.0	91.8	90.2	90.0	92.7
Serine	90.6	96.0	91.8	90.2	90.0	92.7
Tyrosine	86.9	94.6	89.1	88.2	87.7	90.3
Taurine	43.5	71.2	34.3	39.2	23.0	86.4

**Table 4:** The digestible content and digestible intake of the sulfur amino acids, methionine and cysteine, and the total sulfur amino acid system of each experimental diet

	Diet					
	D1	D2	D3	D4	D5	D6 + T
*Calculated digestible SAA and TSAA contents (g/kg)*						
Methionine	8.4	11.1	13.2	16.6	20.2	16.6
Cysteine	2.7	3.3	2.4	2.5	2.2	3.2
∑ *TSAA* [Met + Cys]	11.1	14.5	15.6	19.2	22.5	19.9
∑ *TSAA* [Met]*	11.7	15.2	16.2	19.8	23.0	20.6
*Calculated daily digestible SAA and TSAA intakes (g/kg BW)*						
Methionine	0.21	0.29	0.33	0.44	0.50	0.46
Cysteine	0.068	0.087	0.062	0.066	0.056	0.084
∑ *TSAA* [Met + Cys]	0.28	0.38	0.40	0.50	0.56	0.54
∑ *TSAA* [Met]	0.29	0.40	0.41	0.52	0.57	0.56

### Feeding trial

Juvenile GG were obtained from The Company One Pty Ltd., in Cairns, QLD, Australia, and transported to the Marine and Aquaculture Research Facility (MARF), James Cook University, Townsville, QLD, Australia. Fish were prophylactically treated with formalin (250 ppm for 25 min) upon arrival. Before the start of the feeding trial, GG were allowed to acclimate to a commercial floating pellet (3 mm, Marine Float, Ridley Agriproducts, Australia). Subsequently, 252 fish were individually selected based on body weight (83.93 ± 8.37 g) and stocked in groups of 14 fish into 100-L rectangular tanks in an indoor RAS. Each diet was randomly assigned to three tanks. Fish were fed to apparent satiation once daily at 10:00 AM, and feed intake was recorded daily. The trial was held under controlled conditions at a water temperature of 28.0 ± 0.1 °C and a photoperiod of 11 L:13 D (photoperiod of the season) using overhead LED lamps. Water quality was recorded daily and maintained as follows: pH 7.6 ± 0.1, salinity 31.9 ± 2.7 ppt, ammonia 0.21 ± 0.15 ppm, nitrite-N 0.8 ± 0.6 ppm, nitrate-N 57.7 ± 41.7 ppm, and dissolved oxygen 110.0 ± 21.5% saturation. The experiment was conducted for 38 days.

### Sample collection

Based on unpublished data on gut transit time generated by the aquaculture nutrition laboratory at James Cook University (Townsville, Australia), GGs were last fed 9 h prior to sampling to ensure the timely collection of sufficient fecal material for subsequent digestibility assessment. At the completion of the feeding trial (*d* = 38), groupers were euthanized by an overdose of AQUI-S (Aqui-S New Zealand Ltd., New Zealand) before individually measuring the total length, total weight, viscera, and liver weight of all GG (*n* = 252).

Blood samples were collected from the caudal vein of fish using EDTA (10%)-coated syringes (3 mL, Terumo) and needles (20G × 1.5″, Terumo) (Khor et al. 2021). Blood samples were centrifuged for 5 min (Mini-Centrifuge, 5000 series, Ohaus Frontier, NJ, USA), and plasma was transferred into 1.5-mL micro-centrifuge tubes and stored at −80 °C until analyzed. Additionally, the liver and posterior intestine from four fish per tank were removed and fixed in 10% buffered formalin for the subsequent histological preparation and analysis. Fecal material was collected by dissection from the posterior intestine. Collected fecal samples (*n* = 14) were pooled from fish from the same tank and stored at −20 °C for subsequent digestibility analysis.

### Liver image acquisition and analysis

Freshly harvested livers of 252 fish were photographed in a light box under standardized LED lighting using methods adapted from Trampel et al. (2005) and Fernando et al. (2022). Livers were carefully patted dry before being placed on a white background inside a light box. The light box was made of light-proof aluminum. A A5000 camera (Sony, Japan) with a 16-mm lens was placed in a slot at the top of the light box, creating a perpendicular distance of approximately 800 mm between the camera and samples. Digital images were recorded using fixed exposure settings of ISO-100, 1/60-s shutter speed, a focal length of 16 mm, and a max camera resolution of 19.8 megapixels.

The color assessment methods were adapted from Weller and Westneat (2019) and van Belleghem et al. (2018). The colors and white balance of all photographs were calibrated with a color-checker chart (ColorChecker Passport, X-Rite Inc., USA) that was included in each photograph (Fernando et al. 2022). Image backgrounds, light reflections, and hepatic blood vessels were removed via remove.bg and Adobe Photoshop (Adobe Inc., USA) to reduce bias in the color composition. The average liver color and the color distance based on CIE Lab color space between individual images were compared using R package colordistance (Weller and Westneat 2019).

### Histological assessment: slide preparation and scanning

Fixed liver and posterior intestinal tissues from a total of four fish per tank (*n* = 72) were trimmed to approximately 3 mm, placed into cassettes, then dehydrated through a graded series of 70%, 80%, 85%, 90%, 95%, and 100% ethanol (HistoCore Pearl Tissue Processor, Leica Microsystems Pty Ltd., Australia), and embedded in paraffin wax (HistoCore Arcadia C & H Embedding Center Leica Microsystems Pty Ltd., Australia). Transverse sections (~4 μm) were prepared using a rotary microtome (CUT 4060 model, microTec GmbH, Germany) and mounted onto slides. Liver and intestinal tissues were stained with hematoxylin and eosin (H&E) for quantitative morphometric and cytological evaluations. Additionally, intestinal tissues were stained with a combination of alcian blue (AB) at a pH of 2.5 and periodic acid-Schiff (PAS) to detect neutral (NM; PAS+; magenta), acid (AM; AB+; blue), and mixed (MM; AB+PAS+; purple) mucins from goblet cells. All stained slides were scanned using an automated slide scanner (Aperio LV1 IVD, Leica Microsystems Pty Ltd., Australia) at 40× magnification.

### Histological assessment: liver

**Fig. 1:**
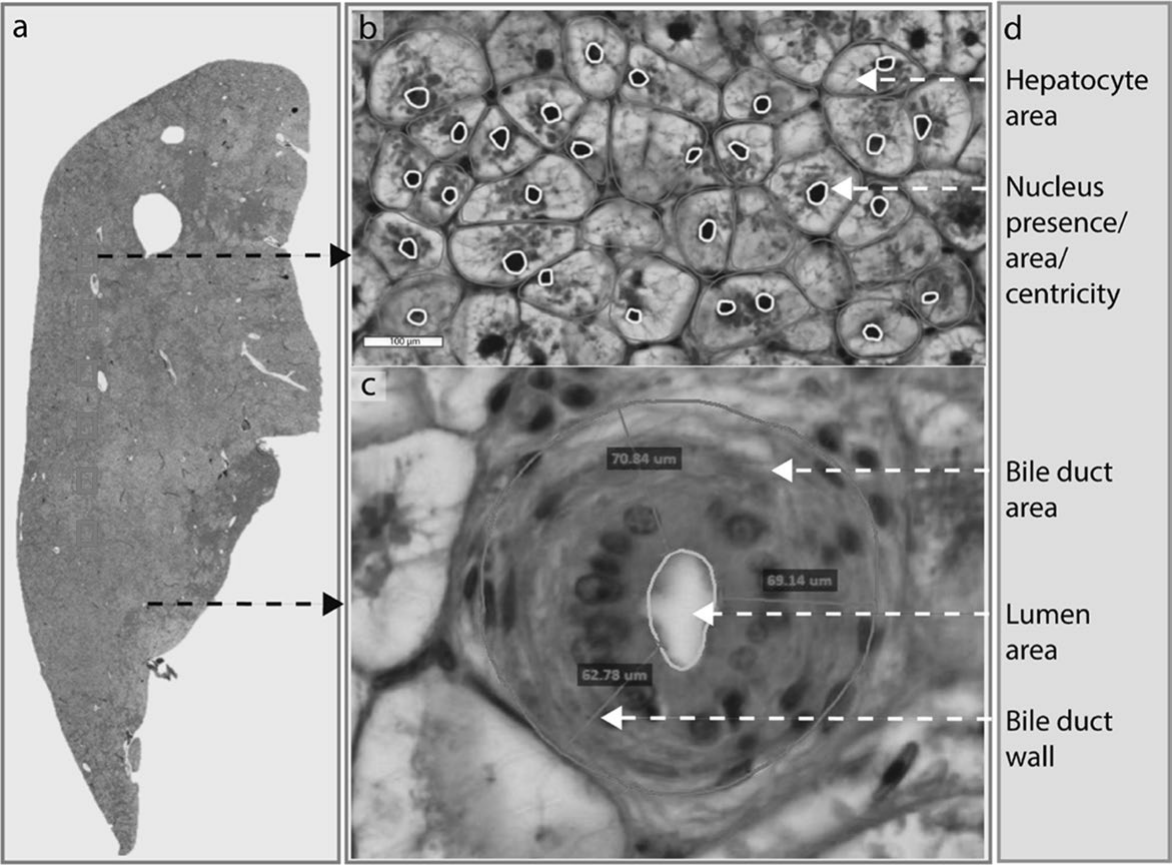
Diagrammatical outline of the manual image analysis of liver cross sections of juvenile giant grouper (Epinephelus lanceolatus) stained with haematoxylin and eosin. (**a**) Whole liver cross section; (**b**) measurements of hepatocyte surface area (μm^2^; pink), nucleus area in (μm^2^; yellow), centricity of nucleus from six intrahepatic fields of interest with a 10× magnification (scale bar = 100 μm); and (**c**) bile duct wall thickness (μm perpendicular to outer wall; pink), bile duct lumen area (μm^2^; blue), and total bile duct wall area (μm^2^; red) from min

Six equally distant uniform segments (40× magnification) of microscopic liver scans per slide were selected on Aperio Image Scope (v.12.4.0.5043) to manually measure individual hepatocyte surface area (μm^2^), assess the presence/absence of nucleus (%), and determine nucleus centricity in (μm^2^) (Fig. 1). Centric nuclei were noted as 2 and non-centric as 1. The same six segments per slide were also used to assess color intensity using Photoshop software. In addition, 6 randomly selected bile ducts per slide were histomorphometrically assessed for bile duct wall thickness (μm), bile duct lumen area (μm^2^), and bile duct wall circumference (μm) using Aperio Image Scope (v.12.4.0.5043) (Fig. 1).

### Histological assessment: posterior intestine

**Fig. 2:**
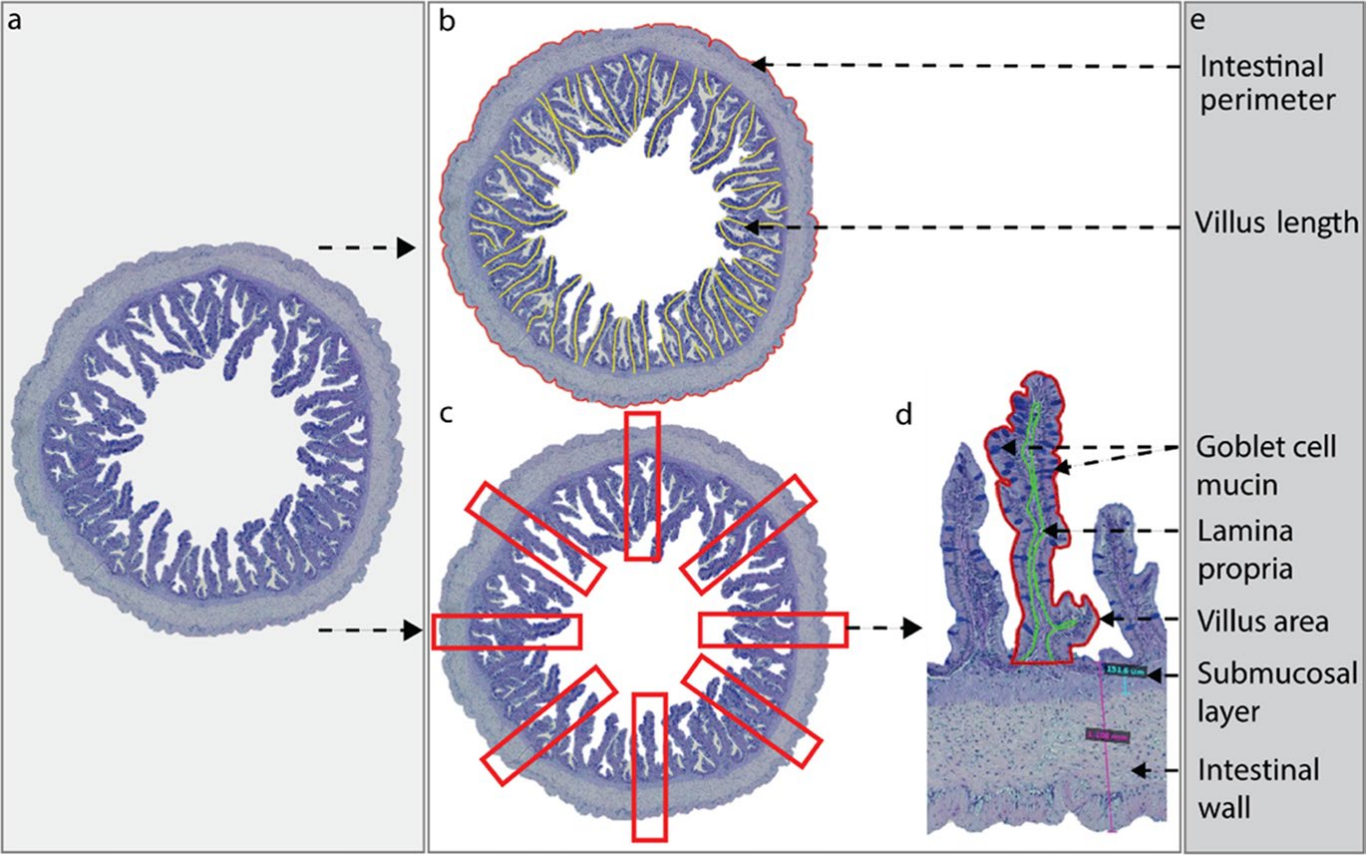
Diagrammatical outline of the manual image analysis of the posterior intestinal cross sections of juvenile giant grouper (Epinephelus lanceolatus) stained with alcian blue and periodic acid-Schiff’s. (**a**) Histological scan of the posterior intestine cross section; (**b**) intestinal circumference (μm) and villus length (μm); and (**c** and **d**) Goblet cell mucin count, lamina propria area (μm^2^), villi area (μm^2^), and intestinal wall thickness (μm) of eight fields of interests of each posterior intestine

All morphometric measurements were performed using Aperio Image Scope (v.12.4.0.5043). For each posterior intestine transverse section stained with alcian blue (AB) and periodic acid-Schiff (PAS), the total intestinal circumference was measured (μm; IC), and the length of each villus was measured by following the center part of the villus (μm; VL) and measured as the distance from the tip of the villus to the stratum compactum (Fig. 2). Eight villi per slide were selected from a total of 72 intestinal slides, according to a structured protocol, starting at the villus closest to the 12 o’clock position and then selecting a further seven that were equidistant around the circular profile of the cross section (Fig. 2). Each villus was measured for villus area (μm^2^; VA) and lamina propria area (μm^2^; LPA). A color adjustment filter was designed (in = 81; out = 208) using Aperio Image Scope to detect and count neutral (NM; PAS+; magenta), acid (AM; AB+; blue), and mixed (MM; AB+PAS+; purple) mucins from goblet cells (Fig. 2).

### Calculations and data analysis

The following calculations were used to determine the relationship between the response variables to varying levels of dietary Met at a constant dietary Cys content and dietary Tau level.

1 $$Weight\ gain\ \left(\%\right)=\left(\frac{Final\ body\ weight- Initial\ body\ weight}{Initial\ body\ weight}\right)\times 100$$

2 $$Specific\;growth\;rate\;\left(SGR;\%/day\right)=\frac{\ln\;Final\;body\;weight-\ln\;Initial\;body\;weight}{Experimental\;period\;\left(days\right)})\times100$$

3 $$Feed\ conversion\ ratio\ (FCR)=\frac{Total\ feed\ intake\ \left(\textrm{g}\ \textrm{DM}/\textrm{fish}\right)}{Weight\ gain\ \left(\textrm{g}\ \textrm{DM}/\textrm{fish}\right)}$$

The apparent digestibility coefficients (*ADCs*) for dietary Met and Cys were calculated to estimate the daily digestible feed intakes, forming independent variables, and were based on the equation reported by Cho et al. (1982), with the exception that yttrium oxide was used as the inert marker (Glencross et al. 2007).

4 $$ADC\;\left(\%\right)=\left(1-\left(\left(\frac{\%Nutrient\;in\;feces}{\%Nutrient\;in\;diet}\right)\times\left(\frac{\%Marker\;in\;diet}{\%Marker\;in\;feces}\right)\right)\right)\times100$$

Digestible feed intake for dietary Met and Cys were calculated as follows:

5 $$\left(Daily\;Met\;intake\;\left(\text{g}\right)\times Met\;ADC\;\left(\%\right)/geom.\;body\;weight\;\left(\text{kg}\right)\right)$$

Two definitions for the requirement of SAA were used: firstly, the *TSAA* [Met + Cys] requirement (National Research Council 2011), if both Met and Cys are present in the experimental diet,

6 $$TSAA\ requirement\ \left[ Met+ Cys\right]= Met\ req.\left(\textrm{g}/\textrm{kg}\right)+ dietary\ Cys\ \left(\textrm{g}/\textrm{kg}\right)$$

, and the calculated *TSAA* [Met] requirement (Ball et al. 2006), if only Met was to represent and meet the SAA requirement; however, an experimental diet with both Cys and Met was used.

7 $$TSAA\ requirement\ \left[ Met\right]= Met\ req.\left(\textrm{g}/\textrm{kg}\right)+\left( dietary\ Cys\ \left(\textrm{g}/\textrm{kg}\right)\times \left( Met\ mol\ weight/ Cys\ mol\ weight\right)\right)$$

### Regression and statistical analysis

Food conversion ratio (*FCR*) and specific growth rate (*SGR*) were used as dependent variables to estimate the dietary, digestible, and daily digestible intake requirements for Met and Cys in juvenile GG. Various non-linear and linear regression models were applied to the data and initially screened via qualities of fit values (*R*^2^, sum of squares, the standard error of estimate), after which the most appropriately fitted models were further cross-validated via the Akaike information criterion (AIC) (Aho et al. 2014). Power series, lognormal, third-order polynomial regressions, and a segmental regression with gentle connection were applied and used to calculate inflection points of the respective response variables, using GraphPad Prism 9.3.1 (471).

Power series:

8 $$Y=A\times {x}^B+C\times {x}^D$$

where *A* and *C* indicate the coefficients and *x*^*B*^ and *x*^*D*^ are the unspecified constants.

The inflection point is calculated as follows:

9 $$X=-{\frac{\left(D\times C\right)}{\left(B\times A\right)}}^{\frac{1}{B-D}}$$

Lognormal:

10 $$Y=\frac{A}{X}\times \exp\ \left(-0.5\times \left(\frac{\ln \left(\frac{x}{GeoMean}\right)}{{\left(\ln (GeoSD)\right)}^2}\right)\right)$$

where *A* is the amplitude and area of the distribution, *GeoMean* is the geometric mean, and *GeoSD* is the geometric standard deviation.The inflection point is calculated as follows:

11 $$X=\exp\ \Big(\ln (GeoMean)\hbox{--} \left(\ln {(GeoSD)}^2\right)$$

Third-order polynomial:

12 $$Y=B0+B1\times X+B2\times {X}^{\hat{\mkern6mu} 2}+B3\times {X}^{\hat{\mkern6mu} 3}$$

where *B* represents each unit by which the slope increases/decreases from the previous unit.

The inflection point is calculated as follows:

13 $$X=\frac{\left(-B3\right)-\sqrt{B{3}^2-\left(3\times B2\times B4\right)}}{\left(3\times B4\right)}$$

All statistical analyses were performed using R version 4.1.1 (R Core Team 2021) using the R packages car, carData, ggplot2, ggpubr, multcompView, and PMCMRplus. Prior to analysis, response variables were validated for assumptions of normality and constant variance via Shapiro-Wilk normality test and Levene’s test for homogeneity of variance, respectively. If assumptions were not met, data were square root or inverse transformed. All response variables were subject to a one-way analysis of variance (ANOVA) defining the effect of five Met levels and two Tau levels. In the event of significance, all six diet’s means were jointly compared via Tukey HSD post hoc test. Effects were considered significant at *P* < 0.05. Data are displayed as mean ± standard error (SEM).

## Results

### Feed and growth efficiency

The feed intake (FI) of GG is strongly dependent on the dietary Met content (Table 5; *P* < 0.05). The increase of dietary Met from 9.5 to 17.9 g/kg at a constant Cys level significantly increased the feed intake in GG until a peak was reached at 152.8 ± 1.0 g/fish (Table 5, Diet 4). At 21.5 g Met/kg (Diet 5), the feed intake significantly decreased to 140.5 ± 0.4 g/fish (*P* < 0.05). The feed intake of GG was not affected by a four-fold increase in Tau, from 1.9 to 8.0 g/kg (Table 5).

**Table 5:** Biometric performance of juvenile giant grouper (Epinephelus lanceolatus) fed one of six dietary treatments, containing each one of five digestible methionine levels and one of two taurine levels (D4: 1.9 g/kg and D6: 8.0 g/kg)

	D1	D2	D3	D4	D5	D6 + T
Digestible Met (g/kg)	**8.4**	**11.1**	**13.2**	**16.6**	**20.2**	**16.6**
Digestible Cys (g/kg)	**2.7**	**3.3**	**2.4**	**2.5**	**2.2**	**3.2**
Initial length (cm)	17.0 ± 0.1	16.9 ± 0.1	17.0 ± 0.1	17.0 ± 0.1	16.8 ± 0.1	16.9 ± 0.1
Initial weight (g)	83.8 ± 1.4	83.7 ± 1.3	83.8 ± 1.2	84.3 ± 1.3	83.8 ± 1.4	84.3 ± 1.2
Final length (cm)	221.0 ± 1.2a	227.1 ± 1.4b	224.8 ± 1.5ab	226.5 ± 1.4ab	224.4 ± 1.4ab	227.1 ± 1.4b
Final weight (FBW; g)	247.9 ± 5.0a	274.4 ± 5.6b	271.6 ± 5.8b	282.0 ± 6.0b	267.7 ± 5.9ab	284.4 ± 5.6b
Weight gain (WG; %)	199 ± 7a	231 ± 8b	225 ± 7ab	237 ± 8b	221 ± 7ab	240 ± 8b
Feed intake (FI; g)	135.5 ± 1.1a	149.0 ± 0.1d	145.0 ± 0.6c	152.8 ± 1.0e	140.5 ± 0.4b	151.7 ± 0.4de
Feed conversion ratio (*FCR*)	0.86 ± 0.03	0.81 ± 0.03	0.81 ± 0.03	0.80 ± 0.02	0.81 ± 0.03	0.81 ± 0.02
Specific growth rate (*SGR*; %/day)	2.83 ± 0.05a	3.10 ± 0.05b	3.07 ± 0.06b	3.15 ± 0.06b	3.03 ± 0.06ab	3.18 ± 0.05b
Hepatosomatic index (HSI)	3.20 ± 0.07a	2.93 ± 0.08bc	2.99 ± 0.05ab	2.86 ± 0.05bcd	2.65 ± 0.05d	2.70 ± 0.05cd

Data are displayed as mean ± standard error (SEM). Different letters indicate significant differences (*P* < 0.05) between diets. *Met*, methionine; *Cys*, cysteine; *Tau*, taurine

Bold entries in this table reflect the digestible methionine and cysteine content (g/kg) of each experimental diet

**Fig. 3:**
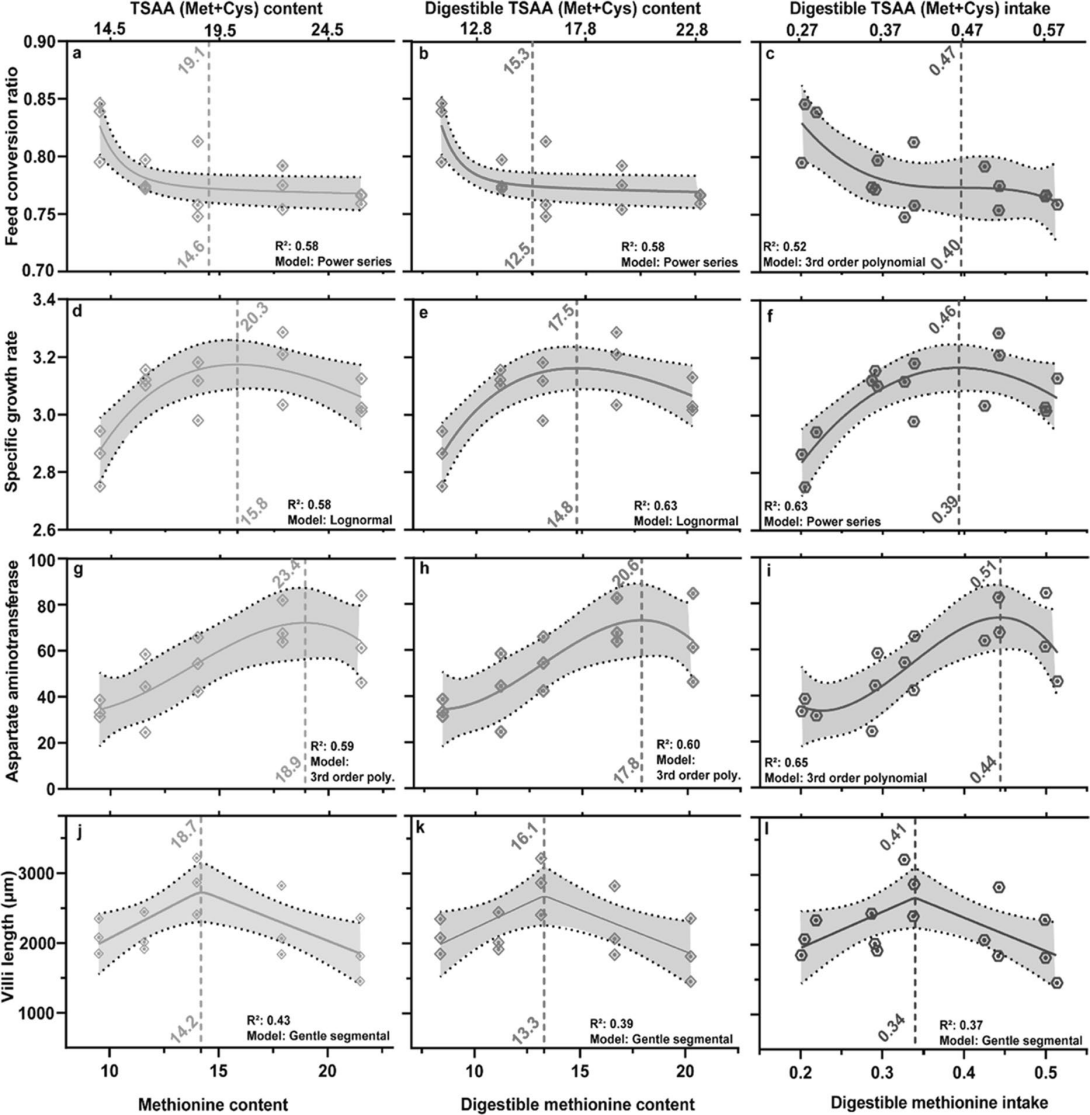
Dose-response curves fitted to *FCR* (**a**, **b**, and **c**), *SGR* (**d**, **e**, and **f**), aspartate transaminase activity — U/L (**g**, **h**, and **i**), and villus length (**j**, **k**, and **l**) in response to five graded level of methionine on a dietary, digestible, and digestible intake basis. The curves and vertical lines (annotated breakpoint) in green identify the breakpoint for dietary methionine (g/kg), bottom *x*-axis, or dietary total sulfur amino acids [Met + Cys] (g/kg), top *x*-axis. The curves and vertical lines (annotated breakpoint) in red identify the requirement for digestible methionine (g/kg), bottom *x*-axis, or digestible total sulfur amino acids (g/kg), top *x*-axis. The blue curves and vertical lines identify the requirement for the daily digestible methionine intake (g/kg BW), bottom *x*-axis, and the daily digestible total sulfur amino acid intake [Met + Cys] g/kg BW, top *x*-axis. Grey semi-transparent areas indicate the 95% confidence interval. Regression models were chosen according to the goodness of fit

**Fig. 4:**
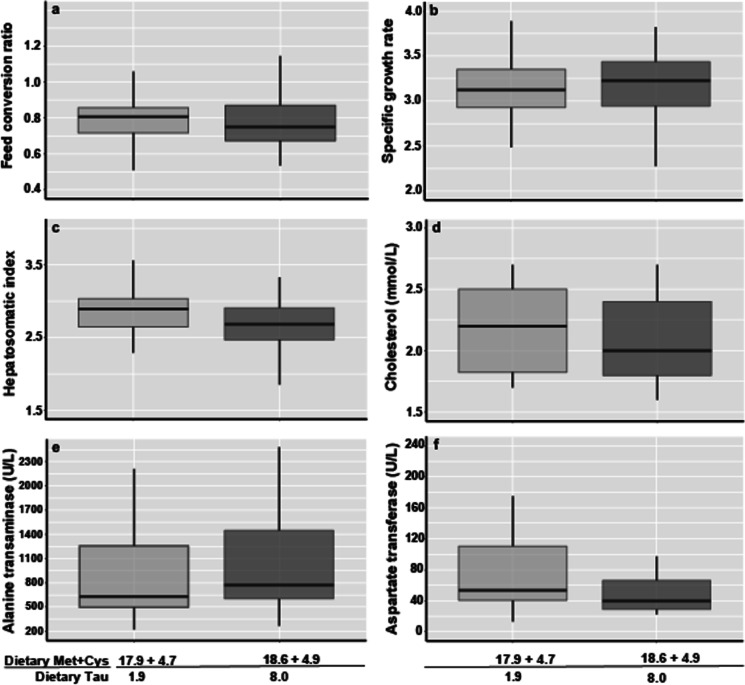
Boxplot on the effects of dietary taurine at 1.9 g/kg (Diet 4) and 8.0 g/kg (Diet 6 + T) at adequate dietary sulfur amino acid levels on **a** feed conversion ratio, **b** specific growth rate, **c** hepatosomatic index, **d** cholesterol, **e** alanine transaminase, and **f** aspartate transaminase in giant grouper (Epinephelus lanceolatus). No significant differences between the dietary treatments were found (P > 0.05)

The *FCR* was not significantly affected by the dietary Met content (*P* > 0.05) nor by a four-fold increase of Tau at a constant level of dietary Met and Cys (Table 5). However, GG fed Diet 1, containing the lowest amount of dietary Met at 9.5 g/kg, had the highest mean *FCR* of 0.86 ± 0.03, whereas GG fed any other dietary level of SAA had a mean *FCR* ranged between 0.80 and 0.81 (Table 5). While *FCR* was not significantly different between diets by ANOVA, the non-linear regression analysis showed that *FCR* decreased with a decrease in dietary Met from 9.5 to 14.6 g/kg, after which the *FCR* did not further decrease (Fig. 3a). The power series model, describing the relationship between the *FCR* and the Met content, had a total sum of squares of 0.005 and an *R*^2^ of 0.58 (Fig. 3a). The power series describing the relationship between *FCR* and the digestible Met intake had a total sum of squares of 0.006 and an *R*^2^ of 0.52 (Fig. 3c). Dietary Tau had no significant effect on the growth performance of GG (Fig. 4b).

Growth parameters (i.e., FBW, WG, and *SGR*) of juvenile GG increased significantly (*P* < 0.05) for all doses of added Met supplementation compared to Diet 1 (Table 5). However, Diet 5 had intermediate values for all growth responses and Diet 3 had an intermediate response for weight gain (Table 5; 1-way ANOVA, *P* < 0.05). An incremental increase in dietary Met at a constant rate of dietary Cys significantly increased *SGR* (*P* < 0.05), peaking in performance at 15.8-g Met/kg diet, after which an increase of dietary Met to 21.5 g/kg led to a decrease in *SGR* (Fig. 3d). The lognormal regression model describing the relationship between *SGR* and Met content had a sum of squares of 0.110 and an *R*^2^ of 0.58 (Fig. 3d). The power series regression model describing the relationship between *SGR* and the digestible Met intake had a sum of squares of 0.098 and an *R*^2^ of 0.63 (Fig. 3f). Suitable models indicated that dietary Met contents between 14.6 and 15.8 g/kg at a constant dietary Cys level of 4.5 g/kg or *TSAA* [Met] contents between 20.1 and 21.3 g/kg optimized the *FCR* and *SGR* of juvenile GG (Fig. 3a and d). In contrast, a digestible Met between 12.5 and 14.8 g/kg at a digestible Cys of 2.8 g/kg or a digestible *TSAA* [Met + Cys] between 15.3 and 17.5 g/kg optimized the *FCR* and *SGR* of juvenile GG (Fig. 3b and e). Lastly, the daily digestible Met intake between 0.39 and 0.40 g/kg BW at a daily digestible Cys intake of 0.07 g/kg BW or a daily digestible *TSAA* [Met] intake between 0.46 and 0.47 g/kg BW optimized the *FCR* and *SGR* of juvenile GG, considering molecular weight difference of Cys and Met (Fig. 3c and f).

### Hepatosomatic index, liver surface color, and histomorphometry

The hepatosomatic index (HSI) of juvenile GG decreased progressively with increasing Met content, with the lowest mean values recorded for GG fed diets with the highest Met content (*P* < 0.05; Table 5). HSI was not significantly affected by a four-fold increase of dietary Tau from 1.9 g/kg (Diet 4) to 8.0 g/kg (Diet 6 + Tau) (Table 5 and Fig. 4c). The digital color assessment of the liver surface color and subsequent color distance assessment via PCoA distribution of Lab color space indicated no dietary Met or Tau clustering. Additionally, there were no significant effects of dietary Met or Tau on the RGB or L^*^a^*^b^*^ liver surface color values of GG (Table 6). The hepatocyte area of GG fed Diet 4 was significantly larger than that of GG fed any other diets (*P* < 0.05; Table 7), whereas GG fed Diet 2 was the smallest. However, no specific trend was observed that indicated a dose-response decrease or increase of the hepatocyte area to the content of dietary methionine (Table 7). No significant differences were observed on nucleus presence and centricity (*P* > 0.05; Table 7). Bile duct wall thickness, bile duct area, and lumen to bile duct area ratio were not affected by dietary methionine or taurine levels in juvenile GG (Table 7).

**Table 6:** Liver surface color of juvenile giant grouper (*Epinephelus lanceolatus*) expressed in RGB and CIE Lab color spaces fed one of six dietary treatments, containing each one of five digestible methionine level and one of two taurine level (D4: 1.9 g/kg and D6: 8.0 g/kg)

	Diet 1	Diet 2	Diet 3	Diet 4	Diet 6 + T
Digestible Met (g/kg)	**8.4**	**11.1**	**13.2**	**16.6**	**16.6**
Digestible Cys (g/kg)	**2.7**	**3.3**	**2.4**	**2.5**	**3.2**
*RGB*					
*R*	91.88 ± 0.60	93.67 ± 0.74	94.77 ± 1.07	94.01 ± 0.96	88.22 ± 0.36
*G*	47.64 ± 0.96	48.28 ± 0.14	47.83 ± 0.53	48.76 ± 1.29	43.74 ± 0.56
*B*	39.83 ± 0.27	40.73 ± 0.59	41.04 ± 0.97	42.33 ± 1.02	37.62 ± 0.36
*Lab*					
*L*	25.16 ± 0.35	25.62 ± 0.09	25.72 ± 0.31	25.83 ± 0.48	23.58 ± 0.20
a^*^	19.16 ± 0.30	19.67 ± 0.34	20.45 ± 0.26	19.7 ± 0.26	19.65 ± 0.19
b^*^	13.95 ± 0.43	14.03 ± 0.51	13.98 ± 0.29	13.28 ± 0.09	13.21 ± 0.02

Data are displayed as mean ± standard error (SEM). Different letters indicate significant differences (*P* < 0.05). *Met*, methionine; *Cys*, cysteine; *Tau*, taurine

Bold entries in this table reflect the digestible methionine and cysteine content (g/kg) of each experimental diet

**Table 7:** Histomorphometry features of juvenile giant grouper (*Epinephelus lanceolatus*) posterior intestine and liver fed one of six dietary treatments, containing each one of five digestible methionine level and one of two taurine level (D4: 1.9 g/kg and D6: 8.0 g/kg)

	Diet 1	Diet 2	Diet 3	Diet 4	Diet 5	Diet 6 + T
Digestible Met (g/kg)	**8.4**	**11.1**	**13.2**	**16.6**	**20.2**	**16.6**
Digestible Cys (g/kg)	**2.7**	**3.3**	**2.4**	**2.5**	**2.2**	**3.2**
*Posterior intestine*						
Intestinal circumference (μm)	45,891 ± 4474	57,753 ± 2928	53,037 ± 3853	48,684 ± 3710	47,135 ± 2254	49,496 ± 4331
Villus length (μm)	3309 ± 85ab	3570 ± 67bc	4319 ± 97d	3795 ± 113c	3595 ± 118a	3146 ± 102bc
Lamina propria/total villus area	0.16 ± 0.01ab	0.18 ± 0.01a	0.14 ± 0.01b	0.14 ± 0.01b	0.15 ± 0.01ab	0.14 ± 0.01b
*Liver*						
Bile duct wall thickness (μm)	93 ± 4	97 ± 4	98 ± 4	93 ± 5	105 ± 4	97 ± 4
Bile duct area (μm^2^)	74,493 ± 12,211	80,531 ± 12,744	76,263 ± 9672	718,489 ± 14,097	85,282 ± 11,383	77,988 ± 14,177
Hepatocyte area (μm^2^)	5363 ± 44bc	4943 ± 43a	5211 ± 38b	6027 ± 53c	5555 ± 48d	5415 ± 45cd
Nucleus presence (%)	55 ± 1	59 ± 1	55 ± 1	55 ± 1	56 ± 1	53 ± 1
Centric nuclei (%)	45 ± 1	41 ± 1	45 ± 1	45 ± 1	44 ± 1	47 ± 1

Data are displayed as mean ± standard error (SEM). Different letters indicate significant differences (*P* < 0.05) between Diet 1 and Diet 6 + T across the respective response variable. *Met*, methionine; *Cys*, cysteine; *Tau*, taurine

Bold entries in this table reflect the digestible methionine and cysteine content (g/kg) of each experimental diet

### Plasma biochemistry

None of the plasma biochemistry response variables in juvenile GG were significantly affected by dietary Met contents (Table 8), except for the aspartate transaminase (AST) activity (*P* < 0.05). Low dietary Met contents of 9.5 g/kg (Diet 1) resulted in significantly lower plasma AST levels (34.4± 5.0 U/L) in comparison to the AST level of 71.3 ± 11.0 U/L of juvenile GG fed diets containing Met at 17.9 g/kg (Diet 4, *TSAA* [Met + Cys] 22.6 g/kg) (Table 8). The plasma AST level of juvenile GG increased significantly from Diet 1 to Diet 4, where Diet 2 and 3 formed intermediate responses for AST (Table 8; 1-way ANOVA, *P* < 0.05). The non-linear regression analysis confirms that an incremental increase in dietary Met at a constant rate of dietary Cys increased AST, peaking in activity at 18.9-g Met/kg diet (Fig. 3g). Thereafter, an increase in dietary Met to 21.5 g/kg led to a decrease in plasma AST activity (Fig. 3e). The 3rd-order polynomial regression model describing the relationship between AST and Met content had an *R*^2^ of 0.59 (Fig. 3e). The 3rd-order polynomial regression model describing the relationship between AST and the digestible Met intake had an *R*^2^ of 0.65 (Fig. 3f). The applied models indicate optimal dietary Met contents of 18.93 g/kg at 4.5 g Cys/kg or a *TSAA* [Met + Cys] content of 23.4 g/kg for maximum AST activity in juvenile GG (Fig. 3e). In contrast, a daily digestible Met intake of 4.43 g/kg BW at a digestible Cys intake of 0.44 g/kg BW or a daily digestible *TSAA* [Met + Cys] intake of 4.88 g/kg BW led to maximum AST performance in juvenile GG (Fig. 3f). Dietary Tau contents of 1.9 g/kg (Diet 4) and 8.0 g/kg (Diet 6 + Tau) did not significantly affect the plasma biochemistry of juvenile GG (Fig. 4d, e, f).

**Table 8:** Plasma chemistry of giant grouper (Epinephelus lanceolatus) fed one of six dietary treatments, containing each one of five digestible methionine level and one of two taurine level (D4: 1.9 g/kg and D6: 8.0 g/kg)

	Diet 1	Diet 2	Diet 3	Diet 4	Diet 5	Diet 6 + T
Digestible Met (g/kg)	**8.4**	**11.1**	**13.2**	**16.6**	**20.2**	**16.6**
Digestible Cys (g/kg)	**2.7**	**3.3**	**2.4**	**2.5**	**2.2**	**3.2**
Albumin (g/dL)	9.9 ± 0.3 (18)	8.9 ± 0.2 (18)	9.3 ± 0.3 (18)	8.4 ± 0.1 (18)	9.2 ± 0.3 (17)	8.7 ± 0.3 (18)
ALP (U/L)	93.9 ± 6.6 (18)	88.5 ± 5.9 (18)	104.5 ± 9.1 (18)	101.2 ± 8.5 (18)	114.0 ± 8.2 (17)	96.1 ± 9.1 (18)
AST (U/L)	34.4 ± 5.0a (18)	42.5 ± 6.5ab (18)	54.2 ± 11.2ab (18)	71.3 ± 11.0b (18)	66.5 ± 11.5ab (17)	61.3 ± 13.3ab (18)
ALT (U/L)	970 ± 129 (18)	836 ± 130 (18)	1125 ± 174 (18)	850 ± 134 (18)	817 ± 147 (17)	907 ± 148 (18)
Cholesterol (mM/L)	2.1 ± 0.1 (18)	2.1 ± 0.1 (18)	2.0 ± 0.1 (18)	2.2 ± 0.1 (18)	2.1 ± 0.1 (17)	2.1 ± 0.1 (18)
Total bilirubin (mg/dL)	2.2 ± 0.1 (18)	2.5 ± 0.1 (18)	2.3 ± 0.2 (18)	2.3 ± 0.1 (18)	2.2 ± 0.1 (17)	2.3 ± 0.1 (18)
Triglyceride (mM/L)	2.1 ± 0.1 (18)	1.7 ± 0.1 (18)	1.8 ± 0.1 (18)	1.9 ± 0.1 (18)	1.9 ± 0.1 (17)	1.8 ± 0.1 (18)
Urea (mM/L)	3.6 ± 0.2 (18)	4.5 ± 0.2 (18)	3.6 ± 0.4 (18)	4.4 ± 0.2 (18)	3.5 ± 0.3 (17)	3.6 ± 0.2 (18)

Data are displayed as mean ± standard error (SEM) and number of fish (*n*). Different letters indicate significant differences (*P* < 0.05). *Met*, methionine; *Cys*, cysteine; *Tau*, taurine; *AST*, aspartate transaminase; *ALT*, alanine aminotransferase; *ALP*, alkaline phosphatase

Bold entries in this table reflect the digestible methionine and cysteine content (g/kg) of each experimental diet

### Intestinal histomorphometry and histochemistry

Intestinal villus length increased with increasing dietary methionine levels, peaking at 14.2-g Met/kg diet, above which it reduced (Fig. 3j). The villi of GG fed Diet 3 (4319 ± 97 μm) and Diet 4 ´(3795 ± 113 μm) were significantly longer than of GG fed diet Diet 1 (3309 ± 85 μm) and Diet 5 (3146 ± 102 μm) (*P* < 0.05; Table 8). The ratio of lamina propria per villus area of juvenile GG significantly decreased with increasing dietary methionine, reaching the lowest ratio from fish fed diet Diet 4, after which it increased (*P* > 0.05; Table 8).

**Fig. 5:**
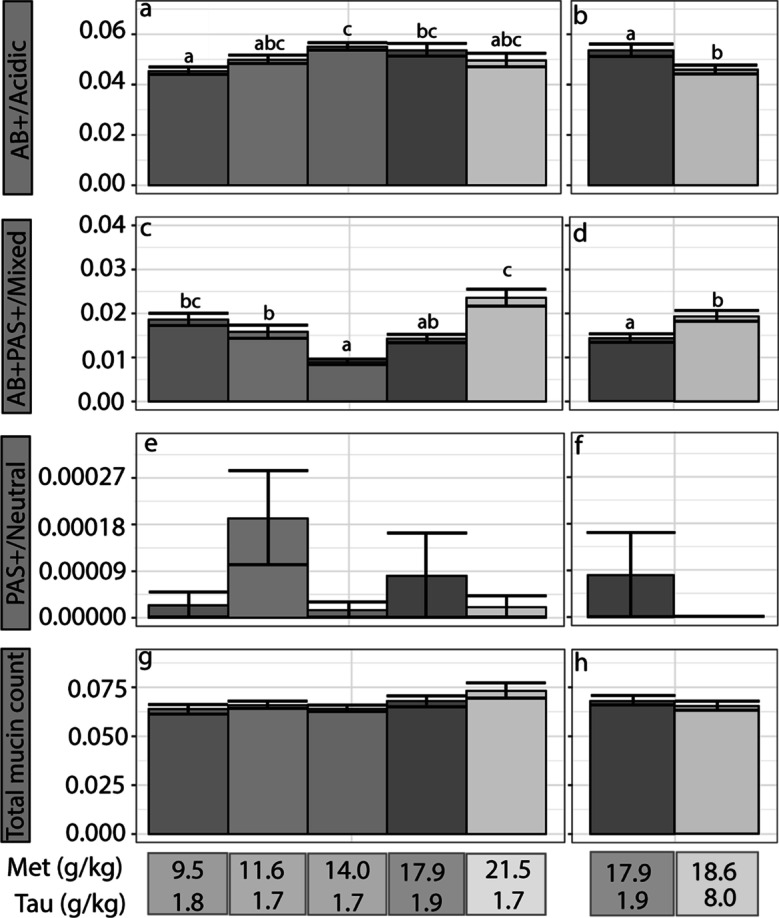
Histochemical analysis (mean ± SEM) of juvenile giant grouper (Epinephelus lanceolatus) posterior intestine, fed one of five methionine levels (**a**, **c**, **e**, and **g**) or one of two taurine levels (**b**, **d**, **f**, and **h**). **a and b** Acidic goblet cell mucin density (AB+ villus mm^2^). **c** and **d** Mixed goblet cell mucin density (AB+PAS+ villus mm^2^). **e** and **f** Neutral goblet cell mucin density (PAS+ villus mm^2^). **g** and **h** Total goblet cell mucin density (TGC/villus mm^2^). Error bars indicate SE

**Fig. 6:**
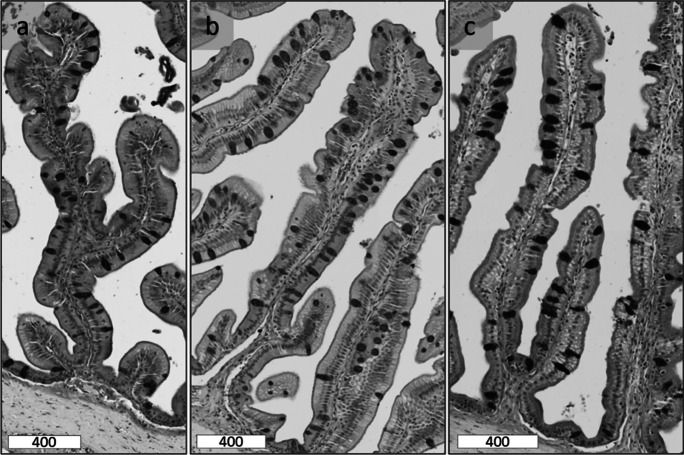
Histological images of the posterior intestinal villi of giant grouper (Epinephelus lanceolatus) fed different dietary treatment groups (stained with alcian blue and periodic acid-Schiff). Acidic goblet cell mucins appear blue due to positive alcian blue staining (AB+). Neutral goblet cell mucins appear magenta due to positive periodic acid-Schiff staining (PAS+). Mixed goblet cell mucins appear purple due to positive alcian blue and periodic acid-Schiff staining (AB+PAS+). **a** Diet 1, showing predominately mixed goblet cell mucin that stained AB+PAS+; **b** Diet 3, showing predominately acidic goblet cell mucin that stained AB+; and **c** Diet 5, showing predominately mixed goblet cell, mucin that stained AB+PAS+. Scale = 400 μm

The density of total goblet mucins was not significantly different in GG fed the different concentrations of dietary Met and Tau (*P* < 0.05; Fig. 5g, h). However, dietary Met and Tau significantly affected the composition of acidic and mixed goblet cell mucins (*P* < 0.05; Fig. 5a–d). The density of acidic goblet cell mucins significantly increased with increasing dietary methionine and peaked when GG were fed Diet 3 (Fig. 5; Fig. 6), where the density then decreased again. In contrast, the density of mixed goblet cell mucins significantly decreased (*P* < 0.05), parallel to the density of acidic goblet cell mucin. The density of mixed goblet cell mucins were the lowest and highest in acidic goblet cell mucins when GG were fed Diet 3 (Fig. 5a–d). The density of neutral goblet cell mucins was low in all GG and was not significantly affected by dietary Met or dietary Tau (Fig. 5e, f).

## Discussion

Multiple studies have shown that Met, Cys, and Tau are metabolically linked nutrients in fish and may alter hepatic and intestinal structures and consequently, functional properties (Candebat et al. 2021; Li et al. 2021). Thus, inadequate provision of essential dietary nutrients is detrimental to optimized growth and feed efficiency. In the present study, SAA requirements of juvenile GG were determined and concentration-specific growth and feed responses to histo-hepatic and histo-intestinal plasticity were mapped. In addition, this study examined whether Tau had ameliorating/enhancing effects on response variables at varying SAA levels.

Our findings indicate that juvenile GG require dietary Met at 14.6 and 15.8 g/kg at a constant Cys level of 4.5 g/kg to optimize growth and feed efficiency, respectively. This finding translates to a dietary *TSAA* [Met + Cys] requirement of 19.1 and 20.3 g/kg and *TSAA* [Met] requirement of 20.1 and 21.3 g/kg in juvenile GG, accounting for the molecular weight difference of Cys (121.16 g/mol) and Met (149.21 g/mol). Other studies determining the dietary Met requirement of *Epinephelus* spp. have found lower or similar SAA requirements than our study. Juvenile hybrid grouper (*E. fuscoguttatus*♀ × *E. lanceolatus*♂) exhibited improved growth at a dietary Met level of 14.5 g/kg at a constant dietary Cys level of 6.9 g/kg (*TSAA* [Met + Cys] requirement = 21.4 g/kg) (Li et al. 2020). In contrast, optimum growth was observed in *E. coioides* at a Met level of 13.1 g/kg and a constant dietary Cys level of 2.6 g/kg (*TSAA* [Met + Cys] = 15.7 g/kg) (Luo et al. 2005). Numerous reasons could affect different *TSAA* requirements, such as species, temperature, and/or animal size. However, Luo et al. (2005) used broken-line regressions to predict breakpoints, which may under-estimate Met requirements, as shown by Shearer (2000). The similarity of SAA requirements between juvenile GG and hybrid grouper (Li et al. 2020), which paternal side is GG, raises the question of whether they may also be similar in other nutrient requirements and whether they share similar nutrient digestibility. To our best knowledge, this study presents the first data on the digestible *TSAA* requirement of GG that considers the apparent digestibility of Met and Cys. The results of our study showed that an apparent digestibility of 88.5 to 94.2% for Met and 53.9 to 77.3% for Cys results in a digestible requirement of Met at 12.5 g/kg to optimize *FCR* and 14.8 g/kg to optimize *SGR* at a constant digestible Cys content of 2.8 g/kg. This results in a digestible *TSAA* [Met + Cys] requirement of 20.3 and 19.1 g/kg, respectively. Further, our results show an apparent daily digestible Met intake requirement of 0.40 g/kg body weight to optimize *FCR* and 0.39 g/kg body weight to optimize *SGR* at a constant digestible Cys intake of 0.07 g/kg body weight. This translates into a daily digestible *TSAA* [Met + Cys] requirement of 0.47 and 0.46 g/kg body weight. These digestible requirements for Met and *TSAA* are lower than those reported by Candebat et al. (2021) for yellowtail kingfish (*Seriola lalandi*). Yellowtail kingfish required a daily digestible Met intake of 0.55 and 0.60 g/kg body weight for optimized *SGR* and *FCR*, respectively, at a constant digestible Cys intake of 0.14 g/kg body weight. Furthermore, GG exhibited depressed growth and feed efficiency when the dietary Met level exceeded the optimum threshold. This growth depression concurs with results from other species, such as yellowtail kingfish (*TSAA* [Met + Cys] ≥ 23.4–26.1 g/kg) (Candebat et al. 2021) and rohu (*Labeo rohita*) (*TSAA* [Met + Cys] ≥ 12 g/kg) (Abidi and Khan 2011). Brosnan and Brosnan (2006) stated that the reduction in growth performance might be caused by the accumulation of toxic end-products of transaminase pathways, such as the neurotoxin, sulfite (Kohl et al. 2019), or reactive oxygen species, homocysteine (Caro et al. 2008; López-Torres and Barja 2008). In addition, nutrient-based formulation facilitates the use of a wider variety of protein sources, ranging in concentration and digestibility of protein and essential amino acids (Booth et al. 2010). The dietary requirement, as well as the upper and lower threshold values of *TSAA*, will help to strategically use Met supplementation for the nutrient-based formulation of grouper feed to optimize the growth performance (Nunes et al. 2014; Salze and Davis 2015; Sampath et al. 2020).

It is largely unassessed how dietary Met below and above the requirement affect the functions of specific tissues such as blood, liver, and intestine in fish. However, linking nutrient intake to physiological homeostasis may be useful in understanding the underlying effects on response variables important to the aquaculture industry (Raskovic et al. 2011). Results from this study on plasma AST levels and liver morphometrics indicated physiological dose-response adaptations to an incremental increase of dietary Met of juvenile GG. AST plasma levels increased with increasing dietary Met and peaked at a digestible dietary Met level (17.8 g/kg), which was supra-optimal for growth and *FCR*. This positive AST/Met correlation is consistent with results found in yellowtail kingfish (Candebat et al. 2021). However, in juvenile yellow catfish (*Pelteobagrus fulvidraco*), increased Met intake resulted in a decrease in AST activity (Elmada et al. 2016). In general, elevated AST levels above the reference range are indicative of pathology caused by cell damage. In mice, elevated AST activity levels above the reference range correlated with increasing dietary Met and hepatitis (Yamada et al. 2012). To date, reference ranges or indications of pathology for AST in GG are largely unassessed. AST is a naturally occurring enzyme in blood plasma as well as other tissues, so plasma AST levels may also be elevated due to increased enzyme activity. In our study, AST levels were elevated in a similar pattern to growth but not for other cell damage markers (i.e., ALT and ALP). Recasens and Mandel (1980) indicated that AST and Cys sulfinate transaminase are used interchangeably in the Tau and glutamate metabolism to reduce either aspartic acid to glutamic acid or cysteine sulfinic acid to beta-sulfinyl pyruvate. Elevated AST levels may therefore indicate the degradation of excess SAA to energy. Throughout this process, Cys is oxidized to Cys sulfinate, which is then deaminated by AST to glutamate, where the remaining carbon skeleton is converted to pyruvate, entering the citric acid cycle (Kohlmeier 2003). Thus, the peak in AST may indicate the maximal reaction rate of AST (18.9 g Met/kg diet) rather than leakage from organ damage. The plasma AST levels in GG peaked at higher dietary Met levels than required for optimal growth or *FCR*, ultimately indicating that the optimal dietary Met and Cys levels for protein synthesis are lower than that which facilitates maximum AST activity.

The liver weight proportional to body weight also referred to as HSI is commonly used in aquaculture nutrition as an indicator of liver function and health (Wang et al. 2005). The incremental increase in dietary Met led to a simultaneous decrease in HSI, which appears to correlate with the increased activity of AST. In fish, the AST activity occurs mainly in the liver, where the Met, Cys, and Tau metabolism also take place (Stipanuk 2020). Although there is limited data on the size of “normal” GG livers, a low-Met diet resulted in a HSI of 3.2. Liver weight is often used in aquaculture as an indication of metabolic competence and health, where lower liver-to-body weight ratios indicates greater liver health and homeostasis (Slooff et al. 1983). Thus, an HSI of 3.2 may indicate adverse nutritional conditions and may be associated with, but not limited to, liver hypertrophy due to lipid, glycogen, or water accumulation or liver hyperplasia (Espe et al. 2010). In parallel with hepatocyte hypertrophy, the color of the liver surface has been reported to change with nutritional conditions, where lipid accumulation may cause increased yellow coloration and glycogen may result in pale livers (Brusle and Anadon 1996; Candebat et al. 2022; Schmitt and Dethloff 2000). However, the results of the present study do not indicate differences in liver surface color or color distance, suggesting that the change in liver weight may instead be associated with hepatocyte hyperplasia rather than hypertrophy. Histomorphometric measurements of hepatocytes and bile ducts revealed no differences between dietary treatments. However, in general, GG hepatocytes appeared to be bloated and larger than that of other species, such as yellowtail kingfish (Liu et al. 2021). In addition, nearly half of the hepatocytes contained no nuclei. Lipid accumulation would have been expected to increase the yellow appearance of the liver surface areas and lightness of the hepato-histological slides due to the wash-out of lipid droplets during the embedding process (Neckel et al. 2016); however, neither was the case in the present study. In salmon, appropriate Met levels reduced the liver size (Espe et al. 2008, 2010), whereas insufficient dietary Met led to an increase in liver weight due to lipid accumulation (Espe et al. 2008, 2010), the latter trend being inconsistent with the present study. Further, nuclei of GG hepatocytes may have been decentralized in the hypertrophic hepatocytes and not been captured during the cross-sectioning in 4-μm slides*.* In GG, neither dietary Met nor Tau affected bile duct wall thickness, which aligns with the growth and feed efficiency results where supplemental Tau did not improve performance. However, it was expected that increasing the dietary Met and Tau would increase the production of Tau-conjugated bile acid, which is less cytotoxic than hydrophobic bile acids (Hohenester et al. 2012).

The absorption of nutrients from the intestinal lumen and the distribution into the circulatory system is mediated by the intestinal epithelial cells that line the villus (Kiela and Ghishan 2016). Increased villus length and surface area have been associated with increased nutrient absorption capacity (Dawood 2021). Posterior intestinal villi were the longest at marginally sub-optimal dietary Met contents (14.2 g/kg) for growth or *FCR*. Therefore, longer villi might have formed to optimize nutrient uptake from marginally nutrient-deficient diets. However, previous studies correlating histology to dietary Met content indicated that intestinal villi are the longest at optimal dietary Met contents (Chen et al. 2014; Gao et al. 2018; Li et al. 2020). In carp, exposure to cold water temperatures resulted in an increased length of intestinal villi and altered micro-morphological structures to compensate for slower digestion (Lee and Cossins 1988). The results of our study suggest that longer intestinal villi are not necessarily better in GG but may rather represent an adaptation to improved intestinal absorption and compensate for sub-optimal nutrition, similar to carp exposed to cold water temperatures.

The intestinal villi contain highly adaptable goblet cells that secrete mucins of different pH levels and regulate the contact of villous epithelial cells with lumen contents. Acidic mucin (AB+/stain blue) is suggested to form a barrier difficult for pathogens to penetrate due to increased viscoelasticity and resisting breakdown by bacterial glycosidase activity, whereas neutral mucin (PAS+/stain magenta) promotes nutrient absorption and neutralizes digestive juices to protect the lamina epithelium from autodigestion (Deplancke and Gaskins 2001; Johansson and Hansson 2016; Leal et al. 2017; Machado-Neto et al. 2013). Mixed goblet mucins (AB+PAS+/stain purple) would therefore enable better absorption of nutrients than acidic goblet mucins to compensate for sub-optimal nutrition, while compromising the pathogenic barrier. Therefore, the elastoviscosity, composition, and secretion rate of intestinal goblet mucins directly regulate nutrient digestion and protection against detrimental factors. In our study, juvenile GG had similar overall levels of intestinal goblet mucins, indicating that Met at sub- and supra-optimal levels will not cause hyperplasia of goblet cells. However, a main finding of our study is that GG fed Diet 3, close to meeting the optimal SAA content, induced the secretion of more acidic and less mixed goblet mucins, whereas sub- and supra-optimal Met levels shifted the production toward more mixed and less acidic goblet mucins. Thus, optimal dietary Met conditions for GG shift the posterior intestinal mucin composition toward a lower pH, which provides more protection from detrimental lumen contents, such as bacteria. An acidic environment in the posterior segment of the intestine is consistent with the assumption that the posterior segment of the intestine is a region of low paracellular permeability because it contains more bacteria, bacterial toxins, and fewer free nutrients compared with the pyloric cecum and anterior intestine, which are the nutrient-absorbing regions (Jutfelt 2011). A deficit in Met may require the relaxation of the barrier to allow for prolonged nutrient absorption, risking the translocation of bacteria from the lumen into the epithelial area. Interestingly, adding Tau to a diet that met *TSAA* requirements only had significant effects on mucin composition and resulted in a shift in production toward less acidic and more mixed goblet cell mucin. Apart from this, Tau did not ameliorate the effects of under- or over-supply of *TSAA*, nor did it increase growth and feed conversion efficiency when *TSAA* requirements were already met.

While Met is universally essential in eukaryotic organisms, Tau is not essential in most organisms as it is metabolized by the unidirectional transsulfuration of its precursors Met and Cys (Brosnan and Brosnan 2006). To our knowledge, only a limited number of studies have examined the interactive effects of dietary Met and Tau and determined the relationship between these two metabolically active nutrients in carnivorous fish and, consequently, often failing to specify the synergistic properties. In fish, adequate Met levels promote the metabolic allocation of Met toward protein synthesis (Urbich et al. 2022). Yet, the metabolic allocation of Cys toward the glutathione or Tau pathway is not well understood. In rats, high levels of Cys increased Tau production as opposed to glutathione and decreases the enzyme Cys sulfinate decarboxylase activity (Bagley and Stipanuk 1995). However, the main pathway to produce Tau in carnivorous fish appears to be rate limited through limited availability of the enzyme Cys sulfinate decarboxylase (Park et al. 2001). Met and Tau are often both classified as indispensable and prevail in aquafeeds for carnivorous fish, which are believed to be unable to meet endogenous requirements due to limited capacity to metabolically derivatize Tau and Met (El-Sayed 2014; Sampath et al. 2020). Lin and Lu (2020) found that juvenile GG fed dietary Tau at 3.57 g/kg diet promoted growth and nutrient digestibility. However, the authors failed to report the Met and Cys contents in the experimental diets and could not indicate if the minimum threshold for the SAA requirement in GG was met. As suggested by Lin and Lu (2020), the synergistic effects of Met and Tau need to be investigated to determine if juvenile GG can meet the requirement for Tau via de novo synthesis from Met. Still, Lin and Lu (2020) did show Tau’s functionality to increase nutrient digestibility in GG. In fish, only limited information is available on the stimulatory effects of dietary Tau on bile acid production. However, in *E. aeneus,* dietary Tau supplementation resulted in hypercholesteremic conditions, indirectly indicating an increase in bile acid production (Koven et al. 2016). In this study, however, cholesterol and triglyceride levels in GG were not affected by a higher dose of Tau in the diets. Moreover, results on the metabolic Met-Tau relationship indicated no effect of a higher dietary Tau supplementation at 8 g/kg on growth or *FCR* when the endogenous requirement for SAA was met. In yellowtail kingfish and hybrid striped bass (*Morone chrysops* × *M. saxatilis*), dietary Tau supplementation are also semi-essential and only required if dietary Met or sulfur amino acid level are deficient (Candebat et al. 2020; Suehs and Gatlin 2021). In contrast, if *TSAA* requirements are not met, Tau supplementation can benefit fish growth and improve feed conversion efficiency due to a provoked Tau requirement. Moreover, Tau can also spare a portion of *TSAA* requirements like Cys, essentially becoming a combined *TSAA* + tau requirement value. Overall, Tau can only be synthesized de novo from SAAs via a limited number of metabolic pathways and is thus best described as a semi-essential nutrient.

The posterior intestinal wall thickness of GG from this study was not affected by the dietary Met treatment, but the intestinal stratum compactum was absent. The stratum compactum is associated with increased intestinal flexural rigidity (Bucke 1971) and is thought to serve as a parasite barrier (Barrett et al. 2021). In salmon, a soy protein-based diet led to inflammatory responses and a thickening of the stratum compactum (Knudsen et al. 2008). The absence of an intestinal stratum compactum is more commonly associated with omnivorous or herbivorous fish, e.g., African catfish (Moawad et al. 2017). GG is carnivorous fish that feeds irregularly, with large prey, and thus, the choice of food in the natural environment may have resulted in a morphological adaptation of the gastrointestinal tract without a stratum compactum in the posterior intestine (Olsson 2011). The exception proves the rule, and the general anatomy of a carnivorous fish’s gastrointestinal tract may not always be universally applicable.

In synopsis, our results show that *TSAA* [Met + Cys] requirements were met at 19.1 and 20.3 g/kg in juvenile GG. Moreover, juvenile GG exhibit intestinal macro- and micro-anatomical plasticity of the liver, AST activity, and posterior intestine in relation to different dietary Met levels. Marginal Met deficiencies appear to be compensated by longer intestinal villi to maximize nutrient absorption. In contrast, an insufficient dietary Met content resulted in a high liver-to-body size ratio, suggesting impaired liver homeostasis. Our study also found that supplemental Tau does not improve growth or *FCR* if sufficient dietary SAAs and a high digestibility of proteins are provided. These data facilitate the formulation of feeds specifically for GG and allow the inclusion of more diverse raw materials to reduce reliance on wild-caught fish toward more sustainable and cost-effective feeds. Findings from this study open directions to further investigate the role of Met and Tau on liver functions in GG, as the results from our study are size and culture specific, and the relevance should be considered.

## Data Availability

All data generated or analyzed during this study are included in this published article (tables and figures).
